# Effect of 25% Sodium Reduction on Sales of a Top-Selling Bread in Remote Indigenous Australian Community Stores: A Controlled Intervention Trial

**DOI:** 10.3390/nu9030214

**Published:** 2017-02-28

**Authors:** Emma McMahon, Jacqui Webster, Julie Brimblecombe

**Affiliations:** 1 Wellbeing and Preventable Chronic Diseases Division, Menzies School of Health Research, Royal Hospital Campus, 105 Rocklands Dr, Tiwi NT 0810, Australia; Julie.Brimblecombe@menzies.edu.au; 2 Centre for Population Health Research, School of Health Sciences, University of South Australia, City East Campus, North Tce, Adelaide SA 5001, Australia; 3 The George Institute for Global Health, The University of Sydney, Camperdown NSW 2000, Australia; jwebster@georgeinstitute.org.au

**Keywords:** salt, sodium, reformulation, bread, sales, Indigenous Australians, population health

## Abstract

Reducing sodium in the food supply is key to achieving population salt targets, but maintaining sales is important to ensuring commercial viability and maximising clinical impact. We investigated whether 25% sodium reduction in a top-selling bread affected sales in 26 remote Indigenous community stores. After a 23-week baseline period, 11 control stores received the regular-salt bread (400 mg Na/100 g) and 15 intervention stores received the reduced-salt version (300 mg Na/100 g) for 12-weeks. Sales data were collected to examine difference between groups in change from baseline to follow-up (effect size) in sales (primary outcome) or sodium density, analysed using a mixed model. There was no significant effect on market share (−0.31%; 95% CI −0.68, 0.07; *p* = 0.11) or weekly dollars ($58; −149, 266; *p* = 0.58). Sodium density of all purchases was not significantly reduced (−8 mg Na/MJ; −18, 2; *p* = 0.14), but 25% reduction across all bread could significantly reduce sodium (−12; −23, −1; *p* = 0.03). We found 25% salt reduction in a top-selling bread did not affect sales in remote Indigenous community stores. If achieved across all breads, estimated salt intake in remote Indigenous Australian communities would be reduced by approximately 15% of the magnitude needed to achieve population salt targets, which could lead to significant health gains at the population-level.

## 1. Introduction

Excess salt intake is one of the main contributors to the high rates of hypertension, cardiovascular disease, renal disease, and premature mortality experienced by many countries around the world [1]. It is estimated that 2.5 million deaths could be prevented globally each year if population salt intakes were reduced to 5 g/day (2000 mg) [1]. Reformulation to reduce salt added during food processing is a cost-effective and sustainable strategy to reduce population intakes [2].

Australian Indigenous peoples currently experience a highly disproportionate rate of chronic disease and premature mortality, with life expectancy ten years lower than the non-Indigenous Australian population [3,4]. Those living in remote areas of Australia have even higher burden of disease and lower life expectancy than those living in non-remote areas [3,4]. The factors driving these disparities are complex, including cultural and social dispossession; considerable socioeconomic disadvantage; poorer access to health services; and high-risk health behaviours such as smoking, physical inactivity, and poor nutrition [5]. The diet of those living in remote Indigenous communities is characterised by very low intake of fruit, vegetables, and whole grains, and excessive intake of salt, sugar, and nutrient-poor discretionary foods [6,7,8,9]. It is likely that even small shifts in dietary intake at the population level could considerably reduce the risk of chronic disease [1,6,10]. We previously modelled dietary change needed to meet dietary guidelines without increasing cost, and found that even with large shifts in modelled dietary intake towards dietary recommendations, estimated sodium intake was still 150% of the upper limit [8], highlighting that changes within food manufacturing are essential to reduce salt intake to a level considered healthy. To address this, the Act on Salt partnership formed in 2014 to investigate opportunities to reduce salt intake in remote Indigenous Australian communities.

Nearly half of all sodium in remote Indigenous communities comes from only three food types [6]. The biggest contributor is discretionary (added) salt (19% of all sodium,), followed by bread (18%, range 13%–25%) and processed meats (9%, range 6%–15%) [6]. We worked with the manufacturer of one of the biggest selling breads in remote Indigenous communities—Bush Oven Outback Bread™ (Bush Oven)—to investigate whether the sodium content could be reduced. Bush Oven bread is primarily distributed to remote Indigenous communities across Australia, and the high fibre white Bush Oven—which currently has 400 mg Na/100 g—represents a considerable proportion of the market share of bread in this population. The manufacturer, Goodman Fielder, developed two levels of reduced salt bread (350 mg sodium/100 g and 300 mg sodium/100 g) without adding or increasing other flavour enhancers or preservatives. After testing that shelf life and microbial quality was retained (internal testing by Goodman Fielder as per standard protocol for recipe formulation), we conducted consumer acceptance testing in one remote Indigenous community. In a triangle test with 62 participants from a remote Indigenous community in Northern Territory, participants were unable to detect a difference between standard bread and reduced salt versions (350 mg or 300 mg/100 g) (*p* > 0.05) [11]. In addition, there were no significant differences in sensory characteristics (appearance, whiteness, flavour, sweetness, saltiness, texture, softness, and overall liking rated a 10-point scale) between standard, 300 mg, or 350 mg sodium breads (*p* > 0.05 using ANOVA) [11].

We calculated that 25% salt-reduction in bread has the capacity to reduce salt intake in remote Indigenous communities by 5%, or an average reduction of 140 mg sodium (0.4 g salt) per day [6]—a reduction that could be clinically significant at the population level [12,13,14]. However, to maintain commercial viability and to maximise the clinical impact, it is important that consumers continue to purchase the lower salt bread. The aim of this study was to investigate whether 25% salt reduction would affect sales of Bush Oven high fibre white bread (study bread) in remote Indigenous Australian communities. The secondary aim was to examine whether this would significantly reduce the total sodium of purchased food and drinks, and to model impact on total sodium of a 25% sodium reduction across a broader range of breads.

## 2. Materials and Methods

### 2.1. Study Design

This study was a non-randomised controlled study examining sales of a reduced salt bread (intervention group) versus the regular salt version (control group) in remote Indigenous Australian community stores. The study included four periods in 2015: baseline (23 weeks, February–July), wash-in (six weeks, July–August), follow-up (12 weeks, August–November), and wash-out (six weeks, November–December). Ethical approval was granted by the Human Research Ethics Committee (HREC) of the Nothern Territory Department of Health and the Menzies School of Health Research (HREC-2014-2311), Central Australian HREC (HREC-14-275), Aboriginal HREC of SA (HREC 04-14-595), Western Australian Aboriginal Health Ethics Committee (HREC-599), and the University of South Australia HREC (04-14-595).

### 2.2. Participants

Remote Indigenous communities are discrete geographical locations dispersed widely across the remote areas of Australia (mostly Central or Top-End Australia) inhabited predominately by Indigenous Australian peoples [15]. Many communities are serviced by only one or two community stores and are distanced from alternative food sources, meaning that these stores provide the majority of dietary intake [16]. Stores are often owned by the community, and decisions regarding the store are made by a committee of community representatives (store committee). There are several store associations that oversee the management of remote community stores; Outback Stores (OBS) and The Arnhem Land Progress Aboriginal Corporation (ALPA) are two of the largest.

Inclusion criteria were remote Indigenous Australian community stores in the Northern Territory, Western Australia, and South Australia managed by either ALPA or OBS, and where the study bread represented ≥45% of the market share for bread (i.e., ≥45% of total dollars spent on bread was on the study bread, assessed using sales data prior to study commencement). Eligible stores were recruited by representatives of ALPA and OBS (in most cases, the area manager or ALPA/OBS nutritionist) who presented the study to store committees and invited them to participate.

This study was initially designed as a randomised controlled trial; however, changes to the baking location and supply route of the bread during the baseline period (for reasons unrelated to this study) meant that half of the participating stores were unable to receive the reduced salt bread. For this reason, the study was adapted to a non-randomised controlled design, with allocation to intervention or control mostly determined by supply route of study bread for participating stores. Stores were blinded to their allocation, and were asked not to discuss the study with customers.

### 2.3. Procedures

At baseline, all stores stocked the regular salt study bread (400 mg Na/100 g) as normal. Following this, intervention stores began to receive the 25% reduced salt study bread (300 mg Na/100 g), while control stores continued to receive the regular bread. All stores were asked to order bread as usual throughout the study, but were asked to use a specific order code during the wash-in and follow-up periods when ordering the study bread (with the code differing between control and intervention stores). The reduced salt bread was distributed to the intervention stores for 18 weeks total, the first six weeks of which were designated as a wash-in period to allow for stores to rotate through their existing stock, with the last 12 weeks considered the follow-up period.

The bread packaging was not distinguishable between the regular and reduced salt breads, except for the barcode. The cartons in which the bread was stored prior to delivery were indicated as regular versus reduced salt by a coloured cross marking. The reduced salt study bread was baked, stored, and distributed using the same processes as the regular salt study bread. Bread was distributed from the Independent Grocers warehouse in Darwin with the two types of bread being stored in different locations within the warehouse to prevent the incorrect bread being picked for shipping.

Weekly store sales data on all food and drinks purchased over the study period were collected for the study duration. Data (product codes, description, units sold, and dollar value) were imported into a purpose-built Microsoft Access database (the Remote Indigenous Stores and Takeaways tool) [17]. All food and drink items were linked to nutrient data by assigning a food identification code using the Australian Food and Nutrient database (AUSNUT 2011–2013) [18].

We contacted all stores via telephone fortnightly from the start of washout to the end of the intervention. During these phone calls, store managers were asked if there had been any issues with delivery, any factors that would affect sales of the study bread, and whether there had been any comments from customers about the study bread. Most remote Indigenous community stores operate with a small staff, and in many instances, it was not possible to contact store managers on the first telephone call attempt. Where telephone calls were unsuccessful, there were additional telephone call attempts, and voice messages were left requesting a return call at a convenient time.

### 2.4. Outcomes and Analyses

Sales-related outcome measures (primary outcomes) were: (1) study bread dollars as a percentage of total dollars spent on food and drink (market share); and (2) average weekly dollars spent on the study bread (weekly dollars). To test if the total sodium of all purchased food and drinks were reduced (secondary outcome), we examined total sodium density per megajoule of energy (sodium density; mg Na/MJ energy).

Analyses were performed using mixed models that included main and interaction terms for period (baseline, wash-in, follow-up, wash-out) and group (control, intervention) and a random intercept for store to account for within-store serial correlation of weekly sales data. *p*-values less than 0.05 were considered statistically significant. The “effect size” was calculated by the difference between the control and intervention group in the change from baseline to follow-up. The main analyses were undertaken using intention-to-treat principle.

We repeated the sodium density analysis to examine the potential change in sodium per megajoule in the following scenarios: (i) if 100% study implementation was achieved (i.e., all bread sold in the follow-up period by intervention stores was reduced salt); (ii) if 25% sodium reduction was also applied to the wholemeal version of the study bread; (iii) if 25% sodium reduction was applied to all breads in the Bush Oven loaf range (high fibre white, wholemeal, and mixed grain); and (iv) if 25% sodium reduction was applied across all breads, regardless of brand.

Comments recorded from phone calls that were related to the quality of the study bread were categorised and reported descriptively. Potential outliers were identified by matching information regarding factors that could affect bread sales (including events in the community, delayed or missed deliveries, or changes to ordering patterns) to anomalies in data.

Sensitivity analyses were performed by repeating the main analyses (i) without outliers and (ii) by dropping individual stores to ensure that results were consistent. All analyses were undertaken using STATA Version 13.1 (StataCorp LLC; College Station, TX, USA).

## 3. Results

Twenty-nine remote Indigenous community stores (24 OBS and five ALPA-managed) met the inclusion criteria, and were invited to participate. Of these, 26 stores consented to the study (21 OBS and five ALPA). Of the 21 OBS-managed stores that consented to the study, nine could not receive the reduced salt bread due to their supply route and were allocated to the control group; the remaining twelve were allocated to the intervention group. All five ALPA-managed stores could receive the reduced salt bread and were alternately allocated to the intervention or control group. All stores that consented to the study completed the study.

The intervention group had more stores in Top-End NT, while control stores were mostly in central Australia (Table 1). There were no significant differences in total weekly dollars or megajoules of all food and drink purchases (mean difference using mixed model $8962, 95% CI −13,020, 30,943, *p* = 0.42; 8985 MJ, −5949, 23,919, *p* = 0.24). During the baseline period, the study bread contributed a mean 13.9% (range for individual stores 9.2%–17.2%) and 12.8% (range 5.1%–19.2%) of the total sodium in all purchased food and drinks for intervention and control stores, respectively. There were no significant differences between control and intervention groups at baseline in market share (mean difference 0.09%, 95% CI −0.98 to 1.17; *p* = 0.86), weekly study bread dollars ($476, −588 to 1540; *p* = 0.38) or total sodium per megajoule (−7 mg Na, −36 to 21; *p* = 0.63) (Table 2).

### 3.1. Main Outcomes

Results of the main analyses are shown in Table 2. We found no significant difference in sales outcomes (percentage market share or weekly average dollars) between control and intervention groups in the change from baseline to follow-up. The effect of all food and drink purchases on sodium density was also not statistically significant. Results across all study periods including wash-in and wash-out are shown in Table S1.

### 3.2. Simulations and Sensitivity Analyses

We achieved a high level of implementation fidelity; all study bread loaves sold during baseline and by the control stores during the follow-up period were regular salt (100% implementation) and 95% of the total combined study bread loaves sold by the intervention stores during the follow-up period were reduced salt. We simulated change in sodium density if 100% implementation was achieved; however, the effect size was not statistically significant (Table 3). In simulations where 25% sodium reduction was applied to all breads in the Bush Oven loaf range (high fibre white, wholemeal, and mixed grain) or across all breads regardless of brand, the effect size reached statistical significance (Table 3).

Results of sensitivity analyses are shown in Supplement 2. Repeating the main analyses without outliers did not impact the results. Repeating the main analyses with individual stores removed did not impact the results for sales-related outcomes (percentage market share and weekly dollars), however the difference in sodium density reached statistical significance when one of the control stores was dropped (−11 mg/MJ; 95% CI −22 to −1; *p* = 0.04).

### 3.3. Comments about Bread

Of the nine planned calls to each of the store managers, we successfully contacted store managers a median of seven times each, with a median of seven phone calls (range six to eight) in the intervention group and seven (range one to eight) in the control group. Thirteen comments were recorded relating to quality from eight individual stores (Table 4). Of these, one comment from an intervention store was related to improved quality and twelve comments were related to decreased quality (six from intervention; six from control stores). There were an equal proportion of stores in each group that reported comments related to reduced quality of the bread (27%; *n* = 4/15 in intervention and *n* = 3/11 in control groups). Most comments came from store managers, except one comment from customers for each group.

## 4. Discussion

We found that 25% salt reduction did not affect sales of one of the top-selling breads in remote Indigenous communities. In addition to no effect on sales, comments from customers did not indicate dissatisfaction with the bread. This is consistent with our consumer acceptance testing, where consumers could not detect the difference between the reduced salt versus regular salt bread [11].

Small incremental sodium reductions (sometimes referred to as step-wise changes) are often preferred by food manufacturers to avoid detection by consumers. Mean sodium levels in bread were decreased by ~40 mg/100 g, or 8.6% over three years in Australia [19], and by 100 mg/100 g or 20% over 10 years in the United Kingdom [20]. However, in a recent meta-analysis, we found that sodium could be reduced by up to 40% in bread (from an average of 692 to 484 mg Na/100 g) in a single step change without affecting consumer acceptance [21]. Quilez and Salas-Salvadó (2015) examined change in sales when salt was reduced in par-baked breads distributed in Spain [22]. In this study, sodium content of nine par-baked breads (delivered to stores frozen and baked on-site to be sold to customers) was reduced by an average of 27.7% from 577 to 417 mg Na/100 g, and potassium citrate was added to partially replace the salt content [22]. Sales in the year following salt reduction (3678 tonnes) were not reduced when compared to the year prior to salt reduction (3577 tonnes). Nor was there an increase in customer complaints. Saavedra-Garcia et al. (2016) also found no reduction in sales when salt was reduced by 20% in a “pan frances” bread sold through a bakery in Peru [23]. Our findings are consistent with these studies, and add to the evidence that considerable sodium reductions in bread can be made in a single step change without affecting commercial viability.

Our study, and the studies by Saavedra-Garcia et al. [23] and Quilez and Salaz-Salvadó [22], did not label the breads as lower salt. Most comments in the present study indicating that the bread was different came from store managers, who were aware of the study, but not of their store’s allocation. These comments came in equal proportions from the control group as the intervention group, indicating that store employees perceived a difference because they were expecting the bread to be different or due to attention bias. There is evidence that labelling foods as no/low/reduced salt can influence expectation and taste perception [24,25]. Liem et al. (2012) found that when soups were labelled “now with reduced salt”, participants expected to like them less than the same soups without the labelling [25]. They found that this effect persisted after tasting the soups, with participants reporting lower liking of the labelled soups than the same soup without the label [25]. As foods perceived as healthier are often perceived as being less palatable, food manufacturers often prefer to use a “stealth health” approach to salt reduction, without labelling the product as reduced salt or otherwise drawing the sodium reduction to consumers’ attention [26,27]. It is likely that adopting this “stealth health” approach was important for maintaining sales.

Reduction to 300 mg Na/100 g would place the Bush Oven high fibre white bread as one of the lowest sodium breads on remote Indigenous community store shelves [6], and the wider Australian market [19,28]. In 2010, the mean sodium content of Australian breads was 435 ± 84 mg Na/100 g in 2010, ranging from 235 to 775 mg Na/100 g [28], while a 2011 estimate indicated a mean sodium content of 415 mg/100 g [19]. By comparison, mean sodium content of breads in the United Kingdom (UK) was 380 ± 70 (range 230–791) mg Na/100 g in 2011 [20]. The UK has achieved this by progressively lowering its target for breads, with the current target of an average of 350 mg/100 g to be achieved by 2017 [29]. The wide range of sodium content in Australian breads, and relatively lower sodium content of breads in the UK, suggest that there is considerable potential for further sodium reductions in bread in Australia.

A secondary aim of this study was to observe the effect on sodium density of all purchased foods and drinks. We projected that reducing sodium in the study bread by 25% would reduce sodium density in purchased foods and drinks by 11 mg Na/MJ. Using an average energy intake of 8.9 MJ/day (based on estimated energy requirements as previously calculated in a similar population [6], and sodium density of purchases of 317 mg Na/MJ (as observed at baseline), this would be equal to a 100 mg/day reduction from 2820 mg/day to 2720 mg/day, or 12% of the reduction needed to reduce sodium to the WHO target. While the values at baseline and follow up in the intervention group correspond with this, the difference from the change in the control group was not statistically significant. It is unlikely that this is due to compensation for the reduced salt bread by increasing the sodium density of other foods purchased, and possibly indicates that we had insufficient power to detect the difference in sodium density.

When we modelled 25% sodium reduction across all breads, significant reductions of −12 (−23 to −1; *p* = 0.03) mg/MJ were estimated, equal to a 3.8% reduction from baseline intake in the intervention group. Using an average energy intake of 8.9 MJ/day, a 12 mg/MJ reduction is equal to approximately 110 mg Na/day, which is 13% of the reduction needed to meet the WHO target. Using an alternative average energy intake of 8.5 MJ/day (as per the remote sample of the Australian Aboriginal and Torres Strait Islander Health Survey 2012–2013) [9], the projected sodium reduction would equal approximately 15% of the reduction needed to meet the WHO target. While a sodium reduction of 3.8% or 110 mg/day may seem small, it has the potential to be clinically significant at the population level [12,13,30]. Cobiac et al. (2010) modelled the health benefits and cost-effectiveness of interventions to reduce sodium intake in the Australian adult population [30]. It was estimated that mandatory reduction of the sodium content of Australian breads (to 450 mg Na/100 g), cereals, and margarines would reduce population sodium intake by 5%, leading to health gains of 110,000 (95% uncertainty index (UI) 53,000 to 180,000) disability-adjusted life years (DALYs) and health care savings of $1.5 (0.7 to 2.8) billion Australian dollars. Even in the voluntary reformulation scenario where sodium intake was reduced by only 0.2%, significant health gains (5300 DALYS, 95% UI 2600 to 9200) and reduction in health care costs (77 (37–140) million AUD) were estimated [30]. Nghiem et al. (2016) modelled salt reduction in bread and found significant health gains of 15,600 quality adjusted life years (95% UI 12,600 to 18,900) and health care savings of $83 million NZD (61 to 110) with only modest reduction in sodium intake of 2.3% or 81.5 mg/day [31]. Salt reduction in bread is a cost-effective intervention that has the potential to reduce sodium intake by small but potentially clinically significant magnitudes in the remote Indigenous Australian population, but more wide-spread reduction across the food supply is needed to meet the WHO target [6].

There are several limitations to this study. It may have been underpowered to detect a difference in the outcomes, particularly sodium density. In sensitivity analysis, there was a significant reduction in sodium density if one control store was dropped. Further, when salt reduction was modelled across all breads, the projected reduction was statistically significant, despite the change in effect size from the original analyses being small. This suggests that the variation in the data may have been larger than the effect size, precluding a significant result. This is less of a concern for the sales-related outcomes; while market share was trending towards a reduction compared to control, average weekly dollars was slightly increased, although neither were statistically significant. Further, sensitivity analyses were consistent in indicating no significant effect on sales; therefore, we are confident in our conclusion that sales were not affected.

A further limitation is that we were unable to randomise the study. Due to this, there was a geographical imbalance between the groups, with more intervention stores located in Top-End Australia, and more control stores located in Central Australia. There is a larger degree of temperature variation in Central Australia compared with the Top-End [32], which may have affected purchasing patterns over the study periods, although this is unlikely to be specifically related to bread purchases. While intervention stores appeared to have higher total food and drink revenue at baseline (indicating they may have been servicing a larger population), this difference was not statistically significant, and the proportion of the study bread sales to total sales (market share) was similar between groups.

Finally, the uniqueness of this population may limit transferability to other settings. Most of the communities in this study had access to only one or two stores from which they could purchase bread; therefore, it would be useful to examine change in sales in an urban setting with easy access to several food vendors and a greater variety of breads from which to choose.

Despite these limitations, our study has several strengths. We tested the bread in a “real-life” setting by replacing the regular product with the lower sodium product on store shelves, rather than conducting testing in a research setting. Our study population included a considerable number of communities spread over a large geographical region with a high proportion of consumers of the study bread. Our main study outcomes were objective, and we collected data on all food and drink sales during the study period, which allowed us to track change in market share of the product and to examine whether sodium reduction was compensated for by increasing sodium density of other purchases. We were also able to determine the impact on sales, an outcome considered of key importance to manufacturers.

## 5. Conclusions

There is considerable potential for further sodium reduction in breads to reduce population salt intakes in remote Indigenous Australian communities. We found that it was possible to reduce salt by 25% in a top-selling bread without affecting sales in remote Indigenous community stores. This adds to the evidence that considerable sodium reductions can be made in bread without jeopardising commercial viability. Twenty-five percent salt reduction across all breads could reduce estimated salt intake in remote Indigenous Australian communities by approximately 15% of the magnitude needed to achieve the WHO target, which has the potential to lead to health gains at the population-level. More wide-spread reductions across the food supply will help to meet the WHO salt reduction target.

## Figures and Tables

**Table 1:** Baseline characteristics.

Characteristic	Control	Intervention
Store Association (%OBS)	82% (*n* = 9/11)	80% (*n* = 12/15)
Location		
Top-End NT	18% (*n* = 2/11)	73% (*n* = 11/15)
Central NT	55% (*n* = 6/11)	0% (*n* = 0/15)
Central South Australia	9% (*n* = 1/11)	0% (*n* = 0/15)
Western Australia	18% (*n* = 2/11)	27% (*n* = 4/15)
Food and drink purchases		
Dollars ($/week)	28,191 (11,495, 44,887)	37,152 (22,854, 51,450)
Energy (MJ/week)	17,049 (5706, 28,392)	26,034 (16,320, 35,747)

NT = Northern Territory; OBS = Outback Stores; Values are percentages or median (interquartile range).

**Table 2:** Study outcomes at baseline and follow up.

Outcome	Group	Baseline	Follow-Up	Effect Size
Market share (%)	Control	4.24 (3.43, 5.06)	4.20 (3.48, 4.92)	−0.31 (−0.68, 0.07) *p* = 0.11
	Intervention	4.34 (3.64, 5.04)	3.99 (3.37, 4.60)	
Dollars ($)	Control	1156 (348, 1965)	1106 (376, 1837)	58 (−149, 266) *p* = 0.58
	Intervention	1632 (940, 2325)	1641 (1015, 2266)	
Sodium (mg Na/MJ)	Control	325 (305, 344)	322 (304, 341)	−8 (−18, 2) *p* = 0.14
	Intervention	317 (300, 334)	307 (291, 323)	

Results are margins (95% confidence intervals) from mixed model analysis with group and period as main and interaction terms. Effect size is the difference between control and intervention groups in change from baseline to follow-up periods. Market share (%) is calculated by dollars as a percentage of all food and drink dollars. Dollars ($) indicates average weekly dollars. Sodium (mg Na/MJ) indicates total sodium per megajoule energy of all foods and drinks purchased.

**Table 3:** Simulated effect size for sodium density (mg Na/MJ energy) if sodium reduction were applied to a wider range of breads.

Scenario	Effect Size (95% CI)	*p*
Observed	−8 (−18 to 2)	0.14
100% Implementation	−9 (−19 to 2)	0.11
25% Reduction in wholemeal	−10 (−21 to 0)	0.06
25% Reduction in all Bush Oven loaves	−11 (−21 to 0)	0.05
25% Reduction in all bread	−12 (−23 to −1)	0.03

Effect size is the difference between control and intervention groups from baseline to follow-up periods in change in sodium density (mg Na/100 g) in total food and drink purchases analysed using mixed model analysis with group and period as main and interaction terms.

**Table 4:** Number of stores in each group where customers or store managers made comments related to quality of study bread.

Comment Type	Control (*n* = 11)	Intervention (*n* = 15)
Improved quality	0	1
Tastes better	0	1
Decreased quality	3	4
Tastes worse	2	2
Shelf life/freshness	1	2
Texture	2	1
Size	0	1

Values may not add up to total where a store has made comments across multiple categories.

## Supplementary Materials

The following are available online at http://www.mdpi.com/2072-6643/9/3/214/s1, Table S1: Outcomes for each of the study periods, Table S2.1: Description of outliers.
