# Supplementary material for: Effect of 25% Sodium Reduction on Sales of a Top-Selling Bread in Remote Indigenous Australian Community Stores: A Controlled Intervention Trial
Source: Nutrients. 2017 Feb 28;9(3):214. doi: 10.3390/nu9030214 (PMC5372877; doi:10.3390/nu9030214)
Supplement: Supplementary file 1 [file nutrients-09-00214-s001.docx]

Supplement 1. Outcomes across all study periods

**Table S1:** Outcomes for each of the study periods

| Outcome | Group | Baseline | Wash-in | Follow-up | Wash-out |
| --- | --- | --- | --- | --- | --- |
| Market share (%) | Control | 4.24 (3.43, 5.06) | 4.16 (3.37, 4.94) | 4.20 (3.48, 4.92) | 4.19 (3.50, 4.88) |
|  | Intervention | 4.34 (3.64, 5.04) | 3.96 (3.29, 4.63) | 3.99 (3.37, 4.60) | 3.92 (3.33, 4.51) |
| Dollars ($) | Control | 1156 (348, 1965) | 1115 (340, 1891) | 1106 (376, 1837) | 1163 (467, 1859) |
|  | Intervention | 1632 (940, 2325) | 1668 (1004, 2332) | 1641 (1015, 2266) | 1430 (834, 2026) |
| Sodium (mg Na/MJ) | Control | 325 (305, 344) | 326 (306, 347) | 322 (304, 341) | 324 (305, 343) |
|  | Intervention | 317 (300, 334) | 325 (308, 342) | 307 (291, 323) | 306 (290, 322) |

Results are margins (95% confidence intervals) from mixed model analysis with group and period as interaction terms. Market share (%) is calculated by dollars as a percentage of all food and drink dollars. Dollars ($) indicates average weekly dollars. Sodium (mg Na/MJ) indicates total sodium per megajoule of all foods and drinks purchased.

Supplement 2: Sensitivity analyses

2.1 Outliers

**Table S2.1:** Description of outliers

| **Outlier #** | **Group** | **Description** |
| --- | --- | --- |
| 1 | Intervention | Large community event in week 18 of baseline with large influx of visitors to the community. |
| 2 | Intervention | Overstock of a competitor bread therefore were stocking minimal amounts of the study bread from week 20 of baseline to week 5 of the intervention. |
| 3 | Intervention | Overstock of study bread therefore did not stock the reduced salt bread until week 6 of the intervention. |
| 4 | Intervention | Received an order of the regular sodium bread during the follow-up period resulting in the regular sodium bread being sold in weeks 2-3 of the intervention |
| 5 | Intervention | Ran out of stock due to a missed delivery in week 9 of the intervention |
| 6 | Control | Ran out of stock in week 7 of the intervention |

| **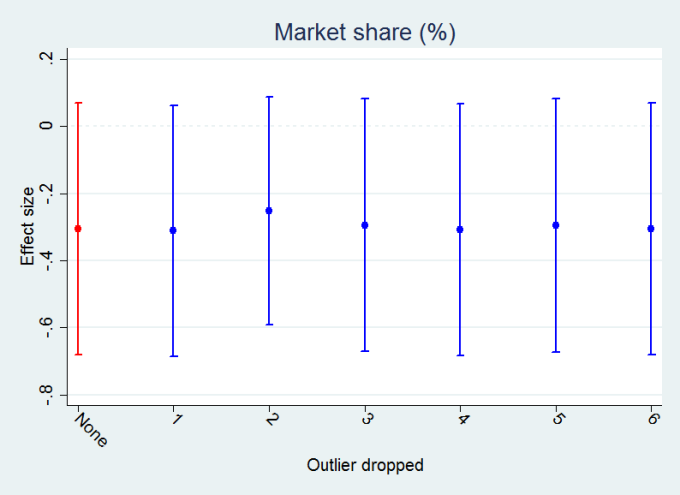(a)** | **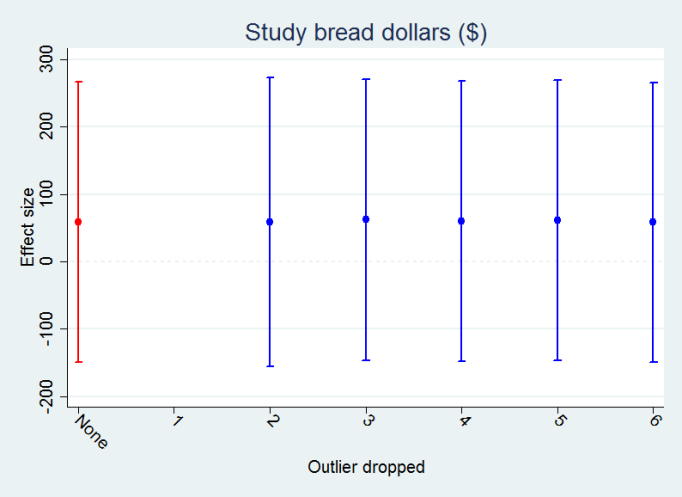(b)** |
| --- | --- |
| **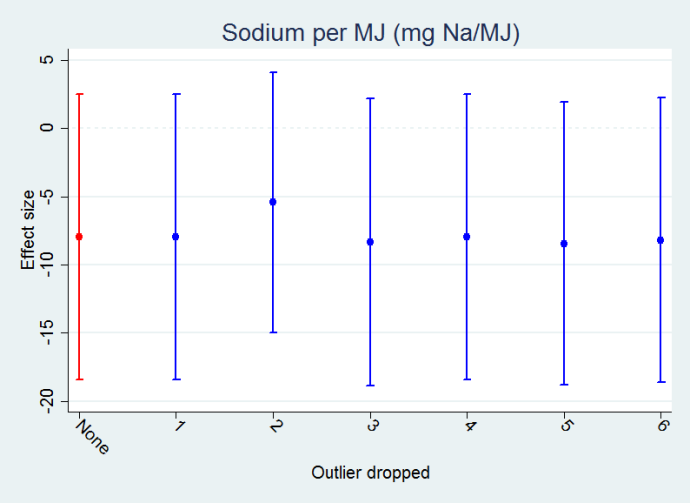(c)** | **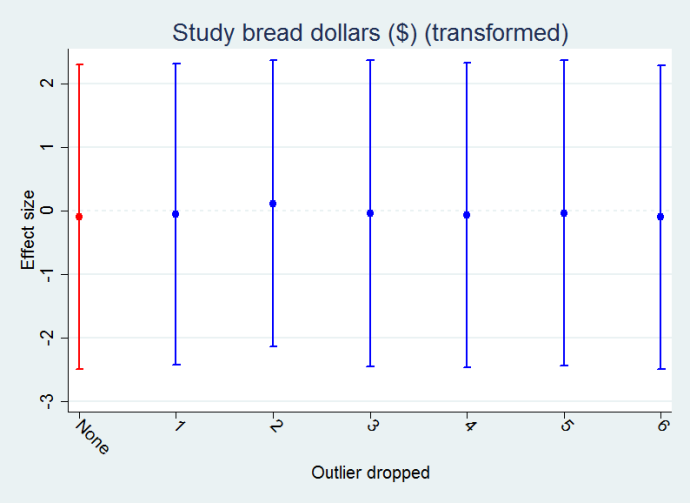(d)** |

**Graph S2.1** Effect size when each outlier is dropped. (a) market share (%) calculated by dollars as a percentage of all food and drink dollars; (b) average weekly dollars spent on study bread; (c) total sodium per megajoule in all food and drink purchases; and (d) average weekly dollars spent on study bread transformed by square root.

Results are effect size (difference between control and intervention groups in change from baseline to follow-up periods) and 95% confidence intervals from mixed model analysis with group and period as interaction terms with each of the outliers dropped (see Table S2).

2.2 Individual stores

| 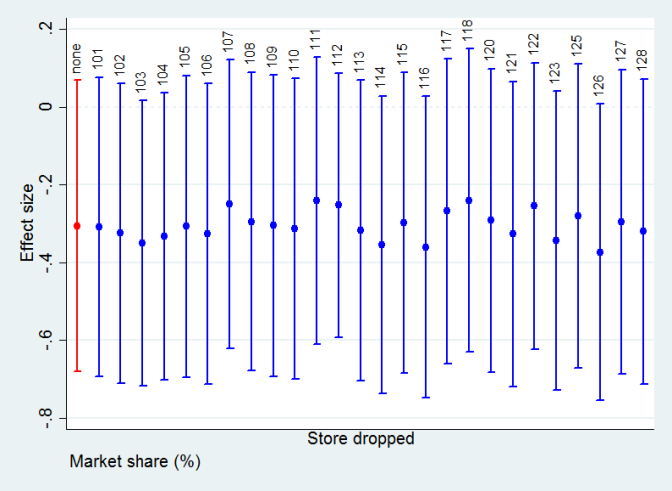 **(a)** | 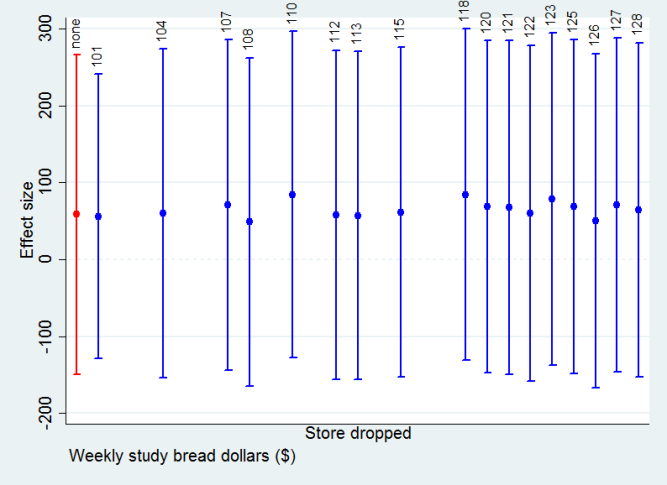 **(b)** |
| --- | --- |
| 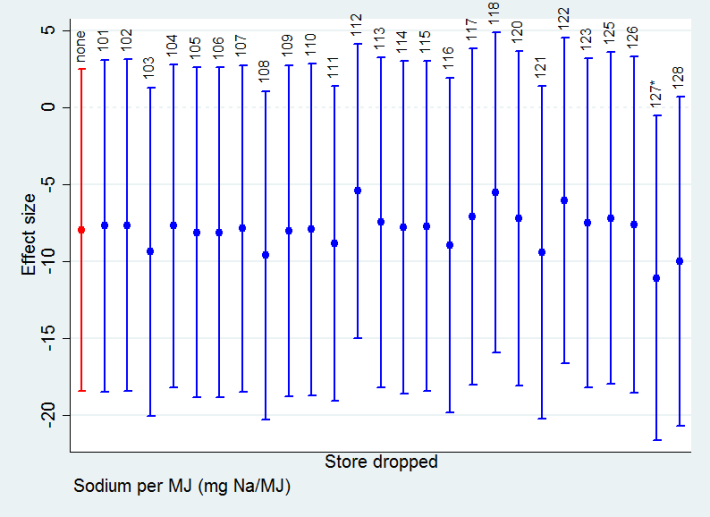 **(c)** | 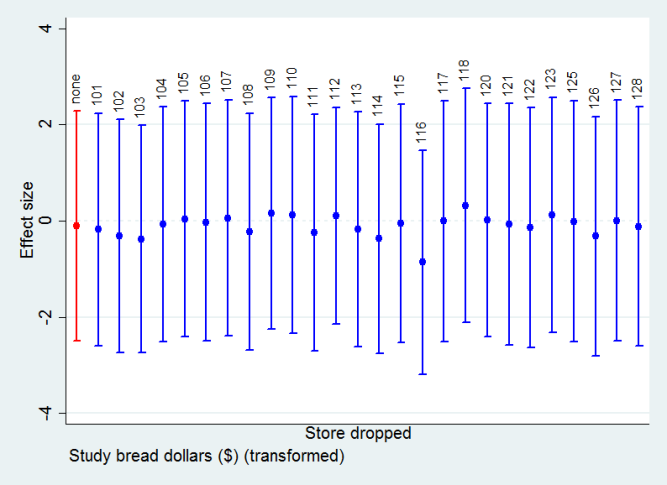 **(d)** |

**Graph S2.2 Effect size when individual stores are dropped** Results are effect size (difference between control and intervention groups in change from baseline to follow-up periods) and 95% confidence intervals from mixed model analysis with group and period as interaction terms when each of the stores is dropped. a) market share (%) calculated by dollars as a percentage of all food and drink dollars; b) average weekly dollars spent on study bread; c) total sodium per megajoule in all food and drink purchases; d) average weekly dollars spent on study bread transformed by square root.
